# NAD-biosynthetic enzyme NMNAT1 reduces early behavioral impairment in the htau mouse model of tauopathy

**DOI:** 10.1016/j.bbr.2017.11.030

**Published:** 2018-02-26

**Authors:** Francesca Rossi, Philippine C. Geiszler, Weina Meng, Matthew R. Barron, Malcolm Prior, Anna Herd-Smith, Andrea Loreto, Maria Yanez Lopez, Henryk Faas, Marie-Christine Pardon, Laura Conforti

**Affiliations:** a School of Life Sciences, University of Nottingham, Queen’s Medical Centre Medical School, Nottingham, NG7 2UH, UK; b Department of Biomedical Sciences, Cagliari University, Cagliari 09042, Italy; c Sir Peter Mansfield Imaging Centre, School of Medicine, University of Nottingham, Queen’s Medical Centre, Medical School, Nottingham NG7 2UH, UK; d Faculty of Medicine, Department of Medicine, Imperial College London, Burlington Danes, Hammersmith campus, London W12 0NN, UK

**Keywords:** Alzheimer’s disease (AD), htau, NMNAT1, NAD, Food burrowing, MRS

## Abstract

NAD metabolism and the NAD biosynthetic enzymes nicotinamide nucleotide adenylyltransferases (NMNATs) are thought to play a key neuroprotective role in tauopathies, including Alzheimer’s disease. Here, we investigated whether modulating the expression of the NMNAT nuclear isoform NMNAT1, which is important for neuronal maintenance, influences the development of behavioral and neuropathological abnormalities in htau mice, which express non-mutant human tau isoforms and represent a model of tauopathy relevant to Alzheimer’s disease. Prior to the development of cognitive symptoms, htau mice exhibit tau hyperphosphorylation associated with a selective deficit in food burrowing, a behavior reminiscent to activities of daily living which are impaired early in Alzheimer’s disease. We crossed htau mice with *Nmnat1* transgenic and knockout mice and tested the resulting offspring until the age of 6 months. We show that overexpression of NMNAT1 ameliorates the early deficit in food burrowing characteristic of htau mice. At 6 months of age, htau mice did not show neurodegenerative changes in both the cortex and hippocampus, and these were not induced by downregulating NMNAT1 levels. Modulating NMNAT1 levels produced a corresponding effect on NMNAT enzymatic activity but did not alter NAD levels in htau mice. Although changes in local NAD levels and subsequent modulation of NAD-dependent enzymes cannot be ruled out, this suggests that the effects seen on behavior may be due to changes in tau phosphorylation. Our results suggest that increasing NMNAT1 levels can slow the progression of symptoms and neuropathological features of tauopathy, but the underlying mechanisms remain to be established.

## Highlights

• First investigation of the role of NMNAT1 in the early stage of tauopathies in mice.
• htau mice display a robust deficit in food burrowing prior to onset of cognitive symptoms.
• NMNAT1 overexpression rescues this behavioral abnormality.

## 1 Introduction

NAD metabolism is increasingly implicated in a variety of biological functions, including regulation of gene transcription, lifespan, cell death, circadian rhythm and glucose homeostasis [1], [2]. The three mammalian isoforms of the central NAD biosynthetic enzyme, nicotinamide mononucleotide adenylyltransferases (NMNATs), have recently emerged as crucial players in neuronal maintenance and protection [3], [4], [5], [6]. They share the same enzymatic activity but a different subcellular distribution [7], [8]. Cytoplasmic NMNAT2 has been recently described as an axon survival factor, a role which is linked to its enzymatic activity, while NMNAT1 appears to have a similar function in neuronal cell bodies [6]. The discovery that loss-of-function mutations in the nuclear isoform NMNAT1 cause Leber Congenital Amaurosis (LCA), a human nervous system disorder characterized by degeneration of retinal neurons and blindness [9], [10], [11], directly links NMNAT1 to neuron survival. Nuclear NMNAT1 has also been associated with neuroprotection in models of human disorders, such as PolyQ toxicity and hypoxic ischemia, with a mechanism correlated to the enzymatic activity and its reported chaperone function [12], [13]. In *Drosophila* a single, homogenously distributed NMNAT isoform has been reported, whose loss causes neuronal and axonal degeneration [3].

Studies in *Drosophila* and mice also highlight NMNAT protective function in models of tauopathies. Neurodegenerative diseases, including Alzheimer’s disease (AD), Pick’s disease and frontotemporal dementia with Parkinsonism linked to chromosome 17 (FTDP-17), are characterized by the presence of hyperphosphorylated tau protein that aggregates and forms neurofibrillary tangles (NFTs) [14]. Tau is a microtubule-associated protein (MAP) expressed in neurons and normally localized in axons, where it promotes microtubule assembly and stabilization, depending on its level of phosphorylation [15], [16]. Conversely, hyperphosphorylated tau, as seen in tauopathies, is unable to stabilize microtubule assemblies and sequesters normal tau and other microtubule stabilizing protein such as MAP1A/MAP1B and MAP2, leading to disruption of microtubules and axonal transport, finally resulting in retrograde degeneration and cell death [17]. Furthermore, abnormally hyperphosphorylated tau leads to the formation of NFTs, which are associated with cellular toxicity and dementia [17]. Overexpression of *Drosophila* NMNAT reduces hyperphosphorylated tau load and rescues cognitive and locomotor deficits caused by overexpression of human wild type or mutant tau in *Drosophila* [18]. Importantly, NMNAT neuroprotection was also confirmed in a mouse model of tauopathy, where NMNAT2 was downregulated in both cortex and hippocampus of mice overexpressing the FTDP-17 associated mutant tau _P301L._ In this model, hippocampal-specific overexpression of either NMNAT1or NMNAT2 significantly reduced hippocampal neuronal loss [19]. Furthermore, it has been recently shown that overexpression of NMNAT1, when redistributed in the cytoplasm (cytNMNAT1), preserves cortical functional connectivity and reduces tau aggregation in the brain of P301S mutant human tau transgenic mice [20]. However, little is known on the role of NMNAT1 in tauopathies, when its nuclear subcellular localization is maintained.

We therefore investigated whether modulating the levels of nuclear NMNAT1 alters the progression of early behavioral dysfunctions and pathological abnormalities, in the htau mouse model of tauopathy which has particular relevance to AD [21] by crossing htau mice with *Nmnat1* transgenic and knockout mice [22], [23].

The htau mouse is characterized by the expression of non-mutant human tau, coding for all six human tau isoforms, including three (3R) and four (4R) microtubule binding domain repeats [21]. This results in an AD-like pathology characterized by early tau hyperphosphorylation, redistribution into cell bodies and dendrites as well as accumulation to form neurofibrillary tangle-like assemblies [21]. We hypothesized that reducing NMNAT1 levels will accelerate, while overexpressing NMNAT1 will slow, the progression of behavioral and neurological features in htau mice. We have extensively characterized the behavioral phenotype of htau mice with aging and found that they display specific early defects in ‘daily life activities’, assessed in the food burrowing test, from after onset of tau hyperphosphorylation[24].

Here we found that total cellular NAD levels were unaltered by NMNAT1 modulation in htau mice although downregulating or overexpressing NMNAT1 significantly reduced or increased NMNAT enzyme activity, respectively. We found that while overexpressing nuclear NMNAT1 reversed early behavioral deficits characteristic of htau mice and attenuated tau hyperphosphorylation, downregulating NMNAT1 had subtle effects on these features and did not cause neurodegenerative changes. Our findings therefore suggest that an increased amount of nuclear NMNAT1 is protective during the early stages of tauopathy in htau mice.

## 2 Materials and methods

### 2.1 Mice and genotyping

All experimental animals used were bred and housed in groups at the University of Nottingham Biomedical Service Unit. The animal work was carried out in accordance with the UK Animals (Scientific Procedures) Act of 1986 (under project licences: PPL 40/3482 and 40/3601), approved by the University of Nottingham Ethical Review Committee and is reported according to the ARRIVE guidelines [25]. All mice were maintained at the University of Nottingham and humanely killed by cervical dislocation. Heterozygous *Nmnat1* knockout (KO) and transgenic mice have been previously described [22], [23]. htau mice (STOCK *Mapt^tm1(EGFP)Klt^* Tg(MAPT)8cPdav/J) were originally purchased from the Jackson Laboratories (Bar Harbor, ME, USA) as described previously [24] and crossed with *Nmnat1* mutant mice to produce experimental htau mice with either increased or reduced levels of nuclear NMNAT1.

Mice were genotyped by PCR using the following primers:

Nmnat1^+/^^−^

Forward 5′-CCCAGTCACTAAGACATTCAA-3′

Reverse 5′-CCTTCTTGCTTCCCACGAGG-3′;

Nmnat1 tg

Forward 5′-ACTTCGGCTCACAGCGCG-3′

Reverse 5′-TCCTTGGCCAGCTCGAACA-3′;

Human tau

Forward 5′-ACTTTGAACCAGGATGGCTGAGC-3′

Reverse 5′-CTGTGCATGGCTGTCCCTACCTT-3′;

Murine tau

Forward 5′-CCAGTTGTGTATGTCCACCC-3′

Reverse 5′-CTCAGCATCCCACCTGTAAC-3′

Disrupted murine tau

Forward 5′-AAGTTCATCTGCACCACCG-3′,

Reverse 5′-TGCTCAGGTAGTGGTTGTCG-3′ [20].

*Nmnat1*^+/−^ and *Nmnat1* tg were crossed with htau mice producing the genotypes of interest that were all heterozygous for murine tau (mtau^+/−^): htau, *Nmnat1* tg/htau, *Nmnat1*^+/−^/htau. Control experiments were first carried out to verify the extent of tau pathology in htau/mtau^+/−^mice. Behavioral data were further collected from relevant control mice (mtau^+/−^, *Nmnat1* tg and *Nmnat1^+/−^* mice), in comparison to wild type mice, as explained below and reported in the supplementary information.

### 2.2 Study design

**Fig. 1:**
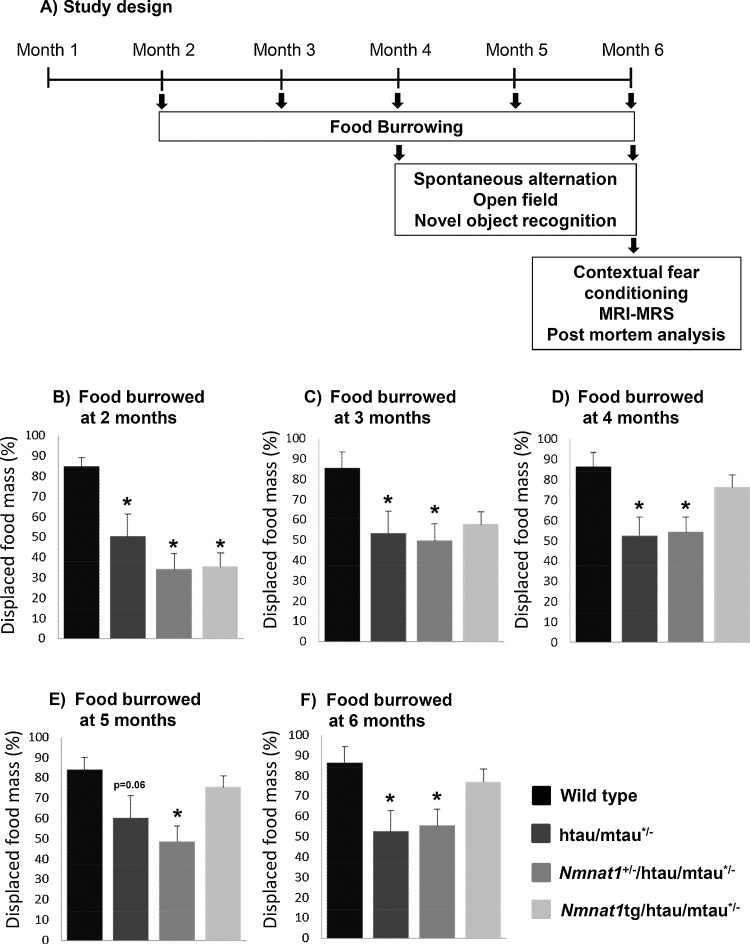
Food burrowing performance in htau/mtau^+/−^ mice is improved by NMNAT1 overexpression. The timeline of *in vivo* assessment is detailed in panel (A). Food-burrowing performance was assessed at two (B), three (C), four (D), five (E) and six (F) months of age. At the age of two months (B), burrowing behavior was strongly impaired in all genotypes compared to wild type control mice. However, from three- to six-months-of-age (C-F), increasing NMNAT1 levels in htau/mtau^+/−^ mice progressively rescued their food burrowing deficit, whilst reducing NMNAT1 levels did not further decrease their low performance. (n = 12/group, three-way ANOVA *, p < 0.05 compared to wild type mice.).

12 mice of each genotype (n = 6/sex) were subjected to a range of tests relevant to AD measuring activities of daily living, learning and memory. Locomotor activity and anxiety-related behavior were also tested. Activities of daily living were measured using the food burrowing test. Memory was assessed using spontaneous alternation in the Y-maze, novel object location/discrimination test and contextual fear conditioning. Open-field was used to test locomotor and anxiety-related behavior. A timeline of the experimental plan and behavioral experiments is illustrated in Fig. 1A. The food burrowing test was repeated from two to six months of age in a monthly interval, the spontaneous alternation and open-field tests were repeated at the age of 4 and 6 months and contextual fear conditioning was performed at the age of six months only, as this test cannot be repeated. Behavioral assessment was followed by *in vivo* magnetic resonance spectroscopy (MRS) and magnetic resonance imaging (MRI) to assess metabolic and structural changes in the hippocampus, after which tissue was collected for post-mortem analyses.

### 2.3 Food-burrowing test

The protocol was adapted from Deacon et al. [26]. Mice were individually placed for one night in a novel cage with *ad libitum* access to food and water. A glass jar containing 30 g of food pellet broken into small pieces was added to the cage. The amount of food displaced from the jar was measured. Data was transformed by square root in order to conform them to a normal distribution [27] and analyzed using 3-way ANOVA with sex and genotype and repeated measures over age, followed by post-hoc planned comparisons.

### 2.4 Spontaneous alternation task

Spontaneous alternation was used to test spatial working memory as previously described [28], [29]. The Y-shaped maze comprised three identical transparent Plexiglas^®^ arms at a 120° angle from each other (41.5 cm in length and 6 cm in width surrounded by 15 cm high transparent Perspex walls). The start point (6 cm x 7.5 cm) was located in the center of the maze, and the animal was allowed to freely explore the three arms over five minutes. The number of alternations was recorded and expressed as a percentage of alternation to estimate spatial working memory performance [28], [29]. Data was analyzed using 3-way ANOVA with sex and genotype and repeated measures over age, followed by post-hoc planned comparisons.

### 2.5 Open-field

Open field was used to assess locomotor and anxiety-related behavior as previously described [29]. Mice were placed in Perspex activity boxes (30 cm × 35 cm × 25 cm) for 30 min during which total distance moved (cm) and percentage of time spent in the center (s) were recorded using Ethovision software (Noldus, Wagenigen, Netherlands). Data was analyzed using 3-way ANOVA with sex and genotype and repeated measures over age, followed by post-hoc planned comparisons.

### 2.6 Novel object location and recognition

Novel object location and novel object recognition were used to measure spatial and recognition memory respectively, as previously described [29]. Briefly, this test consists of three separated trials: in the habituation trial each mouse was allowed to explore two identical objects next to each other along one arena wall. After a 10-min-long interval each mouse was placed into the same arena for the location trial, with one of the two objects being moved across to the opposite wall. After a 20-min inter-trial interval, each mouse was allowed to explore the objects for a third time (discrimination trial) in which one object had been exchanged. Total object exploration times, object location and object discrimination times were recorded using Ethovision software (Noldus, Wagenigen, Netherlands). Data was analyzed using 3-way ANOVA with sex and genotype as between subject factors and repeated measures over age, followed by post-hoc planned comparisons. For each group, preference indices in the object tests were compared to chance levels (50%) using one-sample *t*-tests, in order to determine if the mice were able to discriminate the novel location or novel object.

### 2.7 Contextual fear conditioning (CFC)

Associative learning and memory were assessed using contextual fear conditioning following a previously described protocol [29]. Briefly, this test consisted of three trials conducted on three consecutive days using the same chamber (25 cm × 25 cm × 38 cm): (1) in the acquisition trial, mice were exposed to a series of five foot-shocks (1s-long, 0.4 mA), each separated by a 60-s interval. (2) In the retention trial, mice were placed in the same chamber for one minute without the application of foot shocks. (3) In the one-minute-long extinction trial foot shocks were also omitted. Analysis of the time that the mice spent immobile was conducted in blind twice, and the data were averaged for statistical analysis. Data from the acquisition trial were analyzed using 3-way ANOVA with sex and genotype as between subject factors and repeated measures over time (minutes separating each shock application), followed by post-hoc planned comparisons; data from retention and extinction trials were analyzed using two-way ANOVA, followed by post-hoc planned comparisons.

### 2.8 *In vivo* magnetic resonance spectroscopy and imaging

Animals were anaesthetized with 1% − 1.75% isoflurane in a nitrous oxide:oxygen = 2:1 gas mixture and placed in a custom built holder inside the MR scanner to minimize head motion. Throughout the study, breathing rate and body temperature were monitored continuously and kept constant through adjustment of anesthesia levels and warm water circulation.

*In vivo* MRI and MRS data were acquired from mice of all four genotypes (male and female, age = 6 months, Suppl. Table 1) using a horizontal 7 T MR imaging system (Avance Biospec 70/30 USR; Bruker, Karlsruhe, Germany) equipped with a volume transmit/surface receive radiofrequency coil combination.

MR spectroscopy data were acquired from a 2 × 2 × 2 mm^3^ voxel (volume of interest) centered on the left hippocampus and positioned with multi slice rapid acquisition with relaxation enhancement (RARE) images [30] (Fig. 3A). A line-width of 10–15 Hz for the water peak was obtained after adjustment of first- and second-order shims with FASTMAP [31]. A Point-Resolved Spin echo sequence (PRESS) [32] was used to acquire the MR spectra (repetition time, TR = 2000 ms, echo time, TE = 14 ms, 1024 signal averages) after water suppression with variable pulse power and optimized relaxation delays (VAPOR) [33]. The total acquisition time was 34 min. All spectra processing was conducted blinded to group assignment. Spectra were analyzed using LCModel (Version 6.3-0I) [34]. Resonances corresponding to *myo*-inositol (Ins), taurine, glutamate (Glu), N-acetylaspartate (NAA), glutamine (Gln), glutathione (GSH), glycerophosphocholine and phosphatidylcholine (PCh + GPC), and creatine and phosphocreatine (Cr + PCr) were quantified relative to the total metabolic content (Glu + PCh + GSH + Ins + NAA + Gln + Taurine + Cr + pCr). Only results with the Cramer–Rao lower bounds ≤15% were included in the analysis. Data was further analyzed using ANOVA followed by Bonferroni post-hoc test.

For anatomical MRI, 30 coronal slices were acquired using a RARE sequence (256 × 256 matrix size, field of view, FOV = 1.66 × 1.66 cm, 0.3 mm slice thickness; TR = 7500 ms, effective TE = 29.4 ms, RARE factor = 8, number of averages = 8). The total acquisition time per volume was 32 min. What is referred to as hippocampal areas (covered in 10+/− 1 coronal slices) in the context of this work included fields CA1, CA2 and CA3, the pyramidal cell and lacunosum moleculare layers, the stratum lucidum and radiatum of the hippocampus as well as the dentate gyrus including its molecular, polymorph and granular layers as well as the subiculum relating to Figures. 39 –67 in The Mouse Brain Atlas by Paxinos & Franklin [35]. Hippocampal volumes were determined by manually drawing regions of interest (Fig. 3D) in blind using a software [Medical Image Processing, Analysis and Visualization (MIPAV); v. 7.0.1; Center for Information Technology (CIT), National Institutes of Health (NIH)]. The mean of the hippocampal volume of the left and right hemispheres was expressed as percentage of the total brain volume as outlined in The Mouse Brain Atlas [35]. Since the hippocampal and whole-brain volume did not differ across the two sexes they were combined within each genotype for the statistical analysis of genotype differences. Data was analyzed using ANOVA followed by Fisher’s LSD post-hoc test.

### 2.9 Tissue processing and staining

Mice were humanely killed using an approved Schedule 1 method (cervical dislocation) at six-months-of-age, immediately after the MRS procedure. Half brains from htau/mice, *Nmnat1* tg/htau/mtau^+/−^, *Nmnat1*^+/−^/htau/mtau^+/−^ and mtau^+/−^ mice were post-fixed in 4% PFA for a minimum of 48 h at room temperature. Brains were dehydrated by an ascending alcohol series and then embedded in paraffin. Paraffin blocks were cut into coronal microtome sections (thickness 8 μm) and drawn up on microscope slides.

For evaluation of gross hippocampal morphology, 8 μm coronal sections were mounted on Superfrost slides (BDH), air dried and stained with Haematoxylin and Eosin (H&E). (n = 5/genotype). H&E stained sections were imaged by low power (Plan-NEOFLUOAR 1.25X/0.035) light microscopy (Zeiss Axioplan). Sections were matched between all genotypes and used for the analysis and hippocampal areas were measured using ImageJ software. Data were analyzed using one-way ANOVA followed by Bonferroni post-hoc test.

For immunohistochemistry, cut sections were deparaffinized in xylene and rehydrated through decreasing concentrations of ethanol before the immunohistochemical procedure. Sections were then rinsed in water and subsequently incubated at room temperature in a solution, containing 0.3% H_2_O_2_ diluted in PBS (0.1 mol/L, pH 7.4) for 30 min in order to block endogenous peroxidases. After rinsing, sections were treated with 10 mM sodium citrate buffer pH 6 for 20 min at 100 °C for antigen retrieval. Sections were then incubated with mouse immunoglobulin G blocking reagent (mouse on mouse [MOM] Kit, Vector Laboratories, Burlingame, CA) for 1 h in a humid chamber at room temperature, subsequently incubated with monoclonal antibody CP13 (1:25, generously provided by Prof. Peter Davies, Bronx, NY, USA [21]) and developed with 3,3′-diaminobenzidine (DAB). Sections were scanned and viewed using NanoZoomer digital slide scanner (Hamamatsu, Japan).

### 2.10 Western immunoblotting

Freshly frozen hippocampi and cortices from all genotypes (n = 3-5/genotype) were homogenized in RIPA lysis buffer containing protease and phosphatase inhibitors (Complete mini, Roche). The protein samples were then loaded on a 10% SDS polyacrylamide gel and subsequently transferred to nitrocellulose membrane using a semi-dry blotting apparatus (BioRad). Membranes were blocked for 1 h in 5% BSA in PBS plus 0.2% Tween-20 (PBST), incubated overnight with primary antibody in 5% BSA at 4 °C and incubated for 1 h at RT with HPRT-linked secondary antibody in 3% BSA in PBST. The following primary antibodies were used: mouse anti-synaptophysin (Dako clone Y38, 1:10000), Phospho-PHF-tau pSer202 + Thr205 (AT8, Pierce, 1:500). As loading controls, mouse anti-β-tubulinIII (Sigma, 1:5000) and mouse anti-β-actin (Abcam, 1:5000) were used. Bands were visualized on film using a chemiluminescent detection kit (GE Healthcare). Data was analyzed using one-way ANOVA followed by Bonferroni post-hoc test.

### 2.11 NMNAT enzyme activity assay and NAD level determination

Cortices and hippocampi of 6-month-old mice, collected at the end of the MRS procedure, were snap-frozen in liquid nitrogen and stored at −80 °C. On the day of use, tissues were homogenized in 0.5 M HEPES buffer pH 7.5 and immediately processed for NAD level and NMNAT enzyme activity determination. NAD was then quantified with the HPLC-based assay as described previously [36] (n = 4/group). Data was analyzed using one-way ANOVA followed by Bonferroni post-hoc test.

### 2.12 Control experiments

#### 2.12.1 Tau pathology in htau mice heterozygous for murine tau (htau/mtau^+/−^)

These were compared to control wild type C57BL/6J mice (wild type, Charles River UK). Previous characterization of htau mice has been in the context of the homozygous deletion of murine tau as removal of murine tau was found necessary for the tau pathology to occur in htau mice [21]. However, we have found that murine tau knockout mice develop a behavioral phenotype with aging which bears some similarities to the behavioral phenotype of htau mice but progresses more slowly [24] and, as reported previously, is associated with the occurrence of systemic pathologies [37]. Since there is no evidence that complete removal of murine tau is necessary for the tau pathology to occur in htau mice, we first tested whether a heterozygous murine tau knockout background, which would prevent adverse effects of murine tau deletion, is sufficient for the pathology to occur by carrying out western immunoblotting of total (Tau46), early (CP13) and late (PHF1) pathological stage tau phosphorylation on cortical tissue from three-four 6-month-old mice per genotype, as described previously [24]. These preliminary findings, reported in Suppl. Fig. 1, reveal similar levels of total (Tau46, Suppl. Fig. 1. A&B) and phosphorylated (CP13: Suppl. Fig. 1. C&D; PHF1: Suppl. Fig. 1. E&F) tau in htau mice, regardless of whether they were bred on a full or heterozygous murine tau knockout background. In htau mice, hyperphosphorylated tau was found to redistribute from its axonal position to the cell body and dendrites from 3 months of age [21]. In order to assess whether a similar redistribution was also evident in htau mice heterozygous for murine tau, immunohistochemistry was performed with CP13 antibody [21]. We also found that tau is more abundant in the axons of wild type mice (Suppl. Fig. 1G and I), while it redistributes to a perinuclear location in htau mice which are heterozygous KO for murine tau (htau/mtau^+/−^ Suppl. Fig. 1H and J), similar to previous reports in htau mice homozygous for the murine deletion [21]. These observations indicate that reducing murine tau level by half is sufficient for the tau pathology to develop in htau mice.

#### 2.12.2 Behavioral characteristics of mtau^+/−^, nmnat1 tg and Nmnat1^+/−^ mice

We have previously reported that full murine tau knockout mice (mtau^−/−^) display significant hyperactivity from 4 months of age and deficits in food burrowing from 9 months of age, compared to wild type C57BL/6J mice [24]. We therefore compared the performance of 4- and 6- months-old mtau**^+/−^** and wild type mice (n = 12/genotype) for these two features and found no differences (Suppl. Table 2 and Suppl. Fig. 2A and C). This indicates that potentially confounding effects of murine tau deletion in htau mice can be avoided with a heterozygous murine tau knockout background without affecting the occurrence of tau pathology. Likewise, we assessed these behaviors sensitive to tau dysfunction in *Nmnat1* tg and *Nmnat1^+/−^* mice of the same ages (n = 12/genotype) and found unaltered food burrowing performance (Suppl. Fig. 2B) but *Nmnat1* tg developed an hyperactive phenotype with age (Suppl. Table 1 and Suppl. Fig. 2C).

## 3 Results

The effect of sex/genotype/age interactions and results of the ANOVA for behavioral variables are reported in Table 1.

**Table 1:** Results of the ANOVA tests on behavioral measures in htau/mtau^+/−^ mice.

	Num. df	Den. df	F-value	p-value
Food burrowing test				
sex	1	64	13.96	**<0.001**
genotype	3	64	4.63	**0.005**
age	4	237	13.65	**<0.001**
sex X genotype	3	64	0.37	0.778
sex X age	4	237	0.93	0.449
genotype X age	12	237	1.82	**0.046**
sex X genotype X age	12	237	0.71	0.741

Acquisition of contextual fear				
sex	1	30	0.11	0.737
genotype	3	30	0.04	0.988
minute	4	120	135.69	**< 0.001**
sex X genotype	3	30	0.04	0.987
sex X minute	4	120	1.82	0.13
genotype X minute	12	120	0.4	0.962
sex X genotype X minute	12	120	0.74	0.71

Retention of contextual fear				
sex	1	8.19	0.08	0.775
genotype	3	74.47	0.76	0.527
sex X genotype	3	51.48	0.52	0.67

Extinction of contextual fear				
sex	1	2.05	0.08	0.775
genotype	3	18.62	0.76	0.527
sex X genotype	3	12.87	0.52	0.67

Novel object habituation				
sex	1	64	0.54	0.466
genotype	3	64	0.62	0.603
age	1	64	2.1	0.152
sex X age X genotype	3	64	0.07	0.976
sex X age	1	64	1	0.321
genotype X age	3	64	1.03	0.386
sex X genotype X age	3	64	0.25	0.858

Novel object location				
sex	1	64	0.09	0.771
genotype	3	64	0.3	0.827
age	1	64	7.17	**0.009**
sex X genotype	3	64	0.58	0.633
sex X age	1	64	5.81	**0.019**
genotype X age	3	64	1.11	0.35
sex X genotype X age	3	64	1.06	0.372

Novel object discrimination				
sex	1	64	0.31	0.58
genotype	3	64	1.19	0.319
age	1	64	0.37	0.548
sex X genotype	3	64	1.73	0.17
sex X age	1	64	1.8	0.184
genotype X age	3	64	0.77	0.516
sex X genotype X age	3	64	1.1	0.358

Open field (locomotor activity)				
sex	**1**	64	2.75	0.102
genotype	3	64	5.72	**0.002**
age	1	64	53.11	**< 0.001**
sex X genotype	3	64	2.28	0.088
sex X age	1	64	0.86	0.358
genotype X age	3	64	2.94	**0.04**
sex X genotype X age	3	64	0.7	0.558

Open field (anxiety index)				
sex	1	64	1.42	0.238
genotype	3	64	0.96	0.417
age	1	64	0	0.966
sex X genotype	3	64	0.52	0.668
sex X age	1	64	1.72	0.194
genotype X age	3	64	2.75	**0.05**
sex X genotype X age	3	64	1.95	0.131

### 3.1 NMNAT1 overexpression rescues an early impairment in food burrowing performance in htau mice

Subtle behavioral abnormalities, preceding cognitive dysfunction, are among early signs in AD [38]. In htau mice, cognitive decline has been detected by 12 months of age [39], but we have found earlier non-cognitive behavioral abnormalities in the food burrowing test emerging by 4 months of age, after onset of tau pathology [24]. This test has been used to assess activities of daily living and the inability to perform these activities is an early sign of AD [27]. We therefore subjected the mice to the food burrowing test from 2 months to 6 months of age. There were significant genotype (F_(3.64)_ = 4.63; p = 0.005) and age X genotype interaction (F_(12,237)_ = 1.82; p = 0.046) effects, regardless of sex, but females overall performed better in this task (F_(1.64)_ = 13.96; p < 0.001). htau/mtau^+/−^ mice showed a marked impairment in food burrowing (p < 0.05 compared to wild type at 2, 3, 4 and 6 months of age, Fig. 1B–F). Strikingly, we found that increasing NMNAT1 levels improved the food burrowing performance from 3 months of age and at all later points (Fig. 1C–F), but not at 2 months of age. However, reducing NMNAT1 levels did not further decrease the htau/mtau^+/−^ mice’s impaired performance (Fig. 1B–F). Thus, NMNAT1 deficiency does not contribute to food burrowing impairment in htau/mtau^+/−^ mice, but NMNAT1 overexpression rescues this deficit.

### 3.2 Locomotor activity is increased in htau/mtau^+/−^ mice overexpressing NMNAT1

**Fig. 2:**
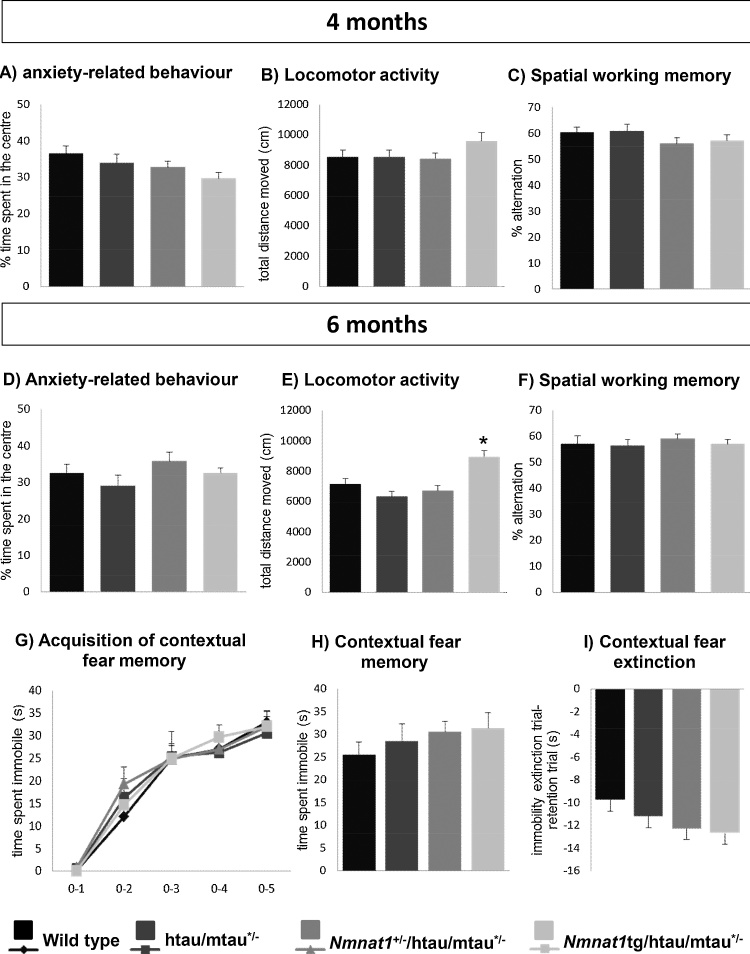
Locomotor activity of 6-months-old *Nmnat1* tg/htau mice is increased. Locomotor activity and anxiety-related behavior were assessed using the open field test while spatial working memory was assessed in the spontaneous alternation test at 4 and 6 months of age. Contextual fear conditioning behavior was evaluated at 6 months of age. As an index of anxiety-like behavior, the proportion of time the mice spent in the center was measured, but no difference was detected between the genotypes at four- (A) and six-months-of-age (D). Locomotor activity, represented as the total distance moved (cm) over 30 min, did not differ between all genotypes at four–months-of-age (B), but was higher in htau/mtau^+/−^ mice overexpressing NMNAT1 at six months-of-age (E). Regardless of age, spatial working memory (C and F), acquisition, retention and extinction of contextual fear (G, H and I) did not differ between genotypes. (n = 12/group, three-way ANOVA; *, p < 0.05 compared to wild type mice).

We assessed locomotor activity and anxiety-related behavior in mice of all genotypes using the open field. Anxiety-related behavior did not differ between the genotypes at both four and six months of age (Table 1 and Fig. 2A and D). Interestingly, there were significant genotype (F_(3.64)_ = 5.72; p = 0.002) and age X genotype interaction (F_(3.64)_ = 2.94; p = 0.04) effects for locomotor activity (Table 1) which was higher in six month old htau/mtau^+/−^ mice overexpressing NMNAT1 than in the other genotypes (Fig. 2A). This age-dependent higher activity is linked to NMNAT1 overexpression as it was also observed in *Nmnat1* tg mice compared to wild type mice (Suppl. Fig. 2D and Suppl. Table 2).

**Fig. 3:**
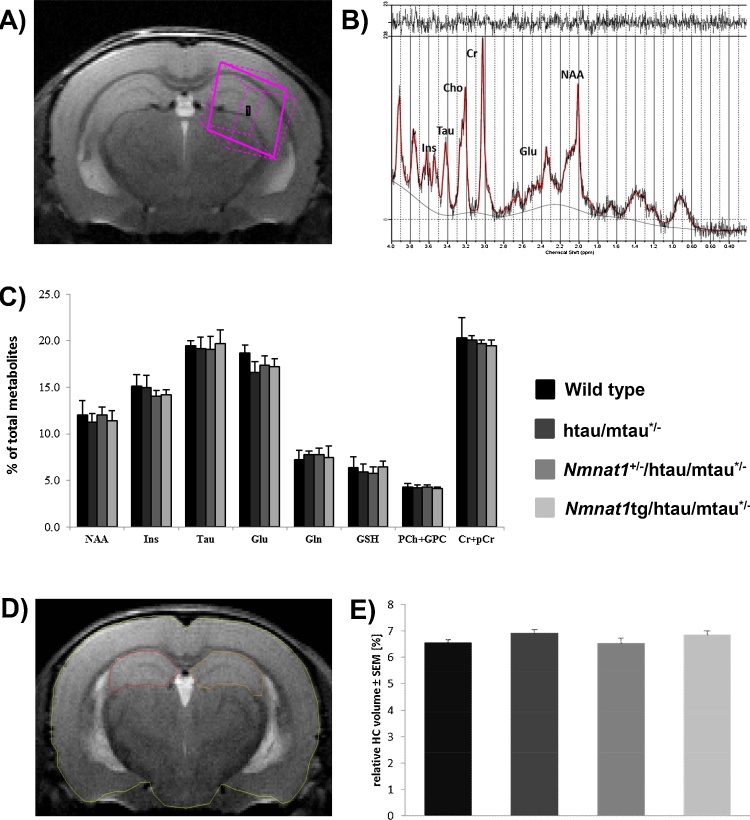
Hippocampal metabolite levels and volume of htau/mtau^+/−^ mice are not altered by modulation of NMNAT1 levels. Relative metabolite concentrations were assessed *in vivo* using MRS with the voxel of interest being positioned in the left hippocampus (2 × 2 × 2 mm^3^) as shown (A). Representative MRS spectrum. Selected metabolites: myo-inositol (Ins), taurine (Tau), glutamate (Glu), N-acetylaspartate (NAA), glutamine (Gln), glutathione (GSH), glycerophosphocholine and phosphatidylcholine (PCh + GPC), and creatine and phosphocreatine (Cr + PCr) (B). Selected metabolite levels were quantified with respect to the total metabolite content as detailed in methods and were unaltered by the genotypes (C). For 3D anatomical MRI, coronal slices were acquired using a RARE sequence and hippocampal volumes were determined in blind by manually drawing regions of interest around the left and right hippocampus, as shown in (D). No differences were detected across the genotypes (E).

### 3.3 htau/mtau^+/−^ mice do not show learning and memory deficits at six-months-of-age

In the spontaneous alternation test, mice of all genotypes alternated arms with a frequency higher than chance without statistically significant differences across the genotypes (Table 1, Fig. 2C and F). Likewise, mice of all genotypes displayed a comparable performance in object recognition and location both at 4 (Suppl. Fig. 3A, C and E) and 6 (Suppl. Fig. 3B, D and F) months, as tested by the novel object paradigm. In the contextual fear conditioning test, during the acquisition trial, all mice were able to acquire contextual fear (F_(4;120)_ = 135.69; p < 0.001. Table 1 and Fig. 2G). No significant differences were detected between the different genotypes in contextual memory retention and extinction (Table 1 and Fig. 2G–H).

### 3.4 Absence of neurodegenerative changes in the htau mouse brain

**Fig. 4:**
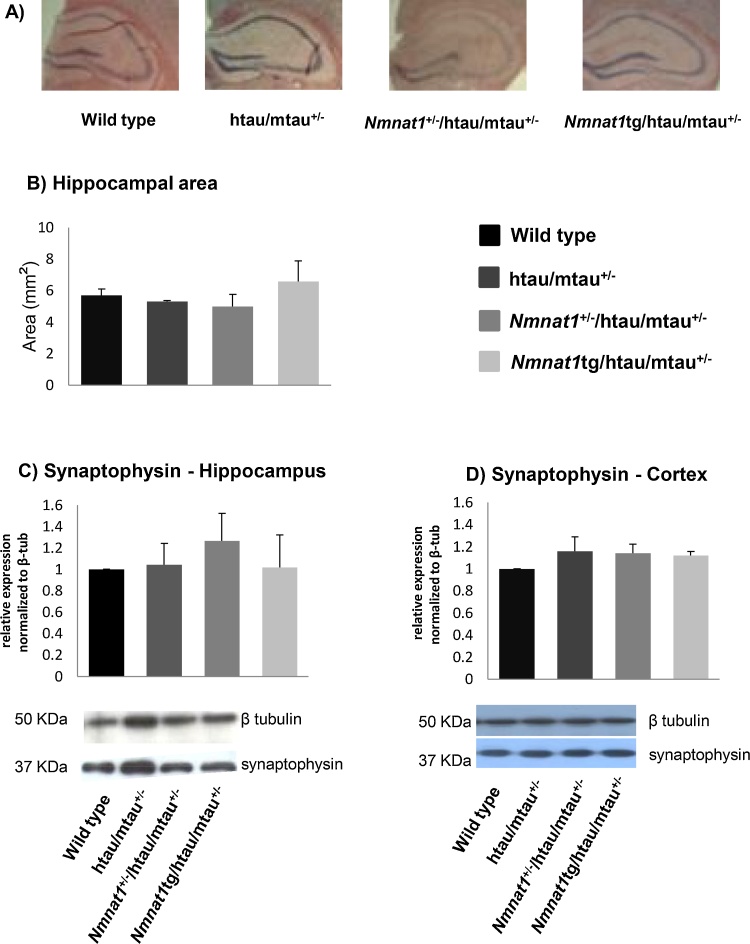
Absence of neurodegenerative changes in the cortex and hippocampus of htau/mtau^+/−^ mice. (A) Coronal sections of brains from all genotypes stained with H-E and (B) relative quantification of the hippocampal areas are shown. Scale bar, 500 μm. There were no differences in the hippocampal area across all genotypes (n = 5/group, one-way ANOVA with Bonferroni post-hoc test, compared to the wild type). (E-F) Western blots of homogenates from hippocampus (C) and cortex (D) were probed with an antibody against the presynaptic marker synaptophysin and with β-tubulin as a loading control. Quantification of the density relative to the loading control showed no difference in the hippocampus (C) and in the cortex (D) (n = 5/group. one-way ANOVA with Bonferroni post-hoc test, compared to control wild type mice).

**Fig. 5:**
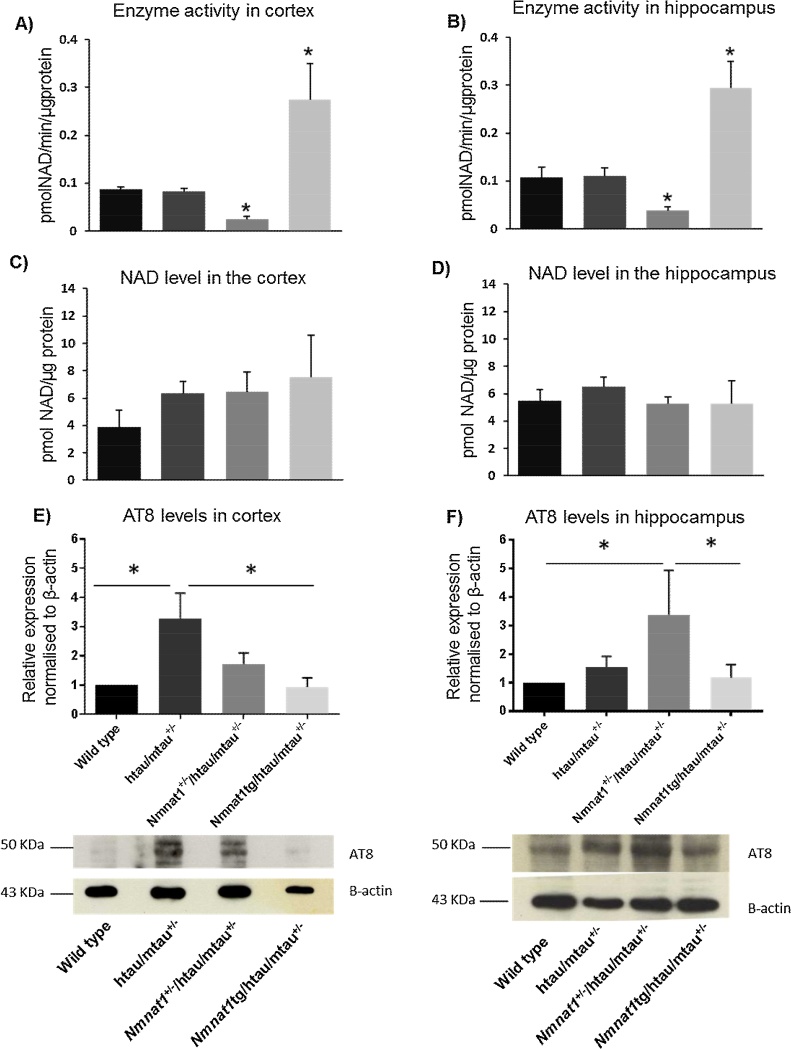
Changes in NMNAT enzyme activity and hyperphosphorylated tau levels. NMNAT enzyme activity (A, B) and NAD levels (C, D) were measured in extracts of cortex (A, C) and hippocampus (B, D). NMNAT1 level modulation changes the enzyme activity but not the NAD levels. Enzyme activity is significantly reduced in *Nmnat1*^+/−^/htau/mtau^+/−^ and increased in *Nmnat1* tg/htau/mtau^+/−^ mice in both the hippocampus and cortex. However, NAD levels did not change by increasing or reducing NMNAT1 in the cortex (C) and hippocampus (D). Levels of hyperphosphorylated tau in cortex of htau/mtau^+/−^ mice appear reduced by *Nmnat1* overexpression. Western blots of cortex (E) and hippocampus (F) homogenates probed with antibody AT8 and their relative quantification indicate that tau immunoreactivity, evident in the cortex of htau/mtau^+/−^ and in the hippocampus of *Nmnat1*^+/−^/htau/mtau^+/−^ mice, are decreased in *Nmnat1* tg/htau/mtau^+/−^ mice (n = 4/group). One-way ANOVA with Bonferroni post-hoc test, compared to wild type mice. *, *p* < 0.05.

We then asked whether any sign of neurodegeneration in the htau/mtau^+/−^ mouse brains could explain the specific defect seen in the food burrowing test (Fig. 1), which has been found to be linked to hippocampal and cortical integrity [40], [41]. First, we measured non-invasively the levels of Ins, taurine, Glu, NAA, Gln, GSH, PCh + GPC, and total creatine (Cr + PCr) *in vivo* using MRS in the hippocampus of six-month-old mice, as the level of these metabolites was found to change during neurodegenerative processes [42], [43]. We did not observe any statistically significant difference in metabolite levels, assessed by MRS across the four genotypes at the time point studied (Fig. 3C). We also measured the volume of the hippocampus *in vivo* (Fig. 3E) and *post-mortem* (Fig. 4A and B) and found no differences across the genotypes. Finally, we determined the levels of the synaptic marker synaptophysin in the hippocampus (Fig. 4E) and cortex (Fig. 5F) and no significant changes were detected in all genotypes.

Taken together, these data show that downregulation of NMNAT1 levels does not cause metabolic or neurodegenerative changes in the brain of htau/mtau^+/−^ mice up to six months of age and that rescue of the early behavioral abnormality, i.e. the reduced performance in the food burrowing test, by NMNAT1 overexpression may not be due to its neuroprotective property.

### 3.5 NAD levels are not affected by changes in NMNAT enzyme activity

We determined NMNAT enzyme activity and NAD levels in the cortex and hippocampus of htau/mtau^+/−^ mice. Overexpression or downregulation of NMNAT1 in htau/mtau^+/−^ mice had expected corresponding effect on enzymatic activity, which was reduced in *Nmnat1*^+/−^/htau/mtau^+/−^ mice and increased in *Nmnat1*tg/htau/mtau^+/−^ mice (Fig. 5A-B). Changes in enzymatic activity were not associated with alterations in steady-state NAD levels (Fig. 5C–D). This indicates that behavioral effects of NMNAT1 overexpression in htau/mtau^+/−^ are not caused by detectable changes in NAD levels.

### 3.6 Levels of hyperphosphorylated tau in brains of htau mice are reduced by NMNAT1 overexpression

We showed previously that deficits in food burrowing develop after onset of tau hyperphosphorylation in htau mice [24]. Therefore in order to test whether behavioral effects of NMNAT1 overexpression are associated with changes in hyperphosphorylated tau, western immunoblotting of AT8 were performed in the cortex (Fig. 5E) and hippocampus (Fig. 5F), as both regions were found to mediate food burrowing performance [40], [41]. AT8 levels were significantly increased in the cortex of htau/mtau^+/−^ mice and this was prevented by overexpressing NMNAT1 in *Nmnat*1tg/htau/mtau^+/−^ mice (Fig. 5 E). In contrast, in the hippocampus, AT8 levels in htau/mtau^+/−^ mice were significantly increased only when NMNAT1 was downregulated (Fig. 5F). These results first suggest that tau pathology develop faster in the cortex than hippocampus, as seen in other models of tauopathy (mtau and rTg4510 lines) [44], [45]. They also suggest that overexpressing NMNAT1 in htau/mtau^+/−^ mice may protect against tau hyperphosphorylation, possibly contributing to alleviation of early behavioral deficits.

## 4 Discussion

NMNAT1 plays an important role in neuron maintenance and its loss of function causes neurodegeneration [11]. In addition, NMNAT1 is neuroprotective in several animal models of neurodegenerative disorders [19]. Our study extends these observations to a mouse model of tauopathy relevant to AD as we found that overexpressing NMNAT1 in mice which express wild type human tau rescues an early behavioral deficit associated with the development of tau pathology [24]. However, cerebral morphometry and cognitive deficits, which are not yet present at this stage of the pathology, were not induced by NMNAT1downregulation. The lack of differences in cognitive deficits is in contrast with previous findings that reported an impairment in visuospatial learning and memory in *Mapt^tm1(EGFP)Klt^* Tg(MAPT)8cPdav/J mouse (htau mice), at six months of age [46]. However, in this pilot study, only three mice were used and tested with different behavioral tests, based on operant conditioning while, in contrast, our tasks aimed to explore spontaneous exploration.

The htau mouse provides a model to study tau pathology relevant to AD through the expression of non-mutant human tau that develops a neuropathology which is reminiscent of that occurring in the early stages of AD [21], [24], [39]. The food burrowing test exploits a natural rodent behavior which requires the integrity of the hippocampus and frontal cortex (brain areas particularly sensitive to AD-like pathology) and is affected following lesions of these brain areas [26], [41]. The first evident symptom in AD is a deterioration in the ability to perform ‘daily-life activity’ which, in mouse models, correspond to burrowing and nest construction [27]. Although behavioral screening in AD patients are mainly focused on cognitive tests, it may be important to assess more specific behaviors which, as suggested by our results, could be modified at early stage. We found that increasing NMNAT1 levels in htau/mtau^+/−^ mice progressively rescued the specific food burrowing deficit at three, four and six months of age, which may be related to a protective effect of NMNAT1 on tau hyperphosphorylation in the cortex. This protective effect might develop with age, which could explain the lack of protection at the first time point tested. Reducing NMNAT1 levels did not further decrease the food burrowing performance of htau mice; this may be due to the fact that the deficit of the htau mouse was already pronounced producing a ceiling effect, or to the fact that NMNAT1 overexpression has a greater impact on NMNAT activity (+200%) than its downregulation (-75%), since baseline NMNAT activity is moderate. However, the fact that NMNAT1 downregulation appear to have opposite effects on tau phosphorylation in the cortex and the hippocampus could also contribute to the lack of exacerbation of burrowing behavior which is mediated by both brain regions [40], [41].

Interestingly, we found that htau/mtau^+/−^ mice overexpressing NMNAT1 retain the significant increase in locomotor activity also evident in six-months-old *Nmnat1* tg mice compared to their wild-type littermates. Overexpression of NMNAT1 has been proposed to delay aging and improve metabolism ([47] and Rossi, Conforti et al., unpublished), an observation that could explain why *Nmnat1* tg mice are more active.

To gain understanding of the mechanisms underlying the modulation of features characteristic of tauopathy in htau mice by alteration in NMNAT1 levels, we compared brain metabolite levels between genotypes *in vivo,* using MRS. This method has been widely used to study cellular mechanisms preceding complex pathological conditions [48] such as in models of amyloidosis and Pick disease [49], [50]. Changes in specific metabolites may indicate the onset or the course of specific neurodegenerative processes, therefore measuring brain metabolism might be a sensitive method to screen for early biomarkers of pathology. For example, NAA, a neuronal marker, is reduced in the medial temporal lobe in AD patients that show only mild cognitive impairments [51]. Taurine, which plays a role in osmoregulation and neuroprotection in the central nervous system, is also reduced in a murine mouse model of amyloidosis [52]. Several lines of evidence suggest that other metabolites, such as myo-inositol, a glial marker, Glu, Gly, and choline, are changed in AD [53], [54]. In the present study, the metabolic contents of the hippocampus in six month old htau/mtau^+/−^ mice remained unchanged. Likewise, no gross structural changes were detected in the presence of human tau as determined by *in vivo* MRI and *post-mortem* tissue. However, these sensitive methods were able to detect changes in other more aggressive models of tauopathies [55] and the lack of changes is therefore consistent with the lack of aggravation of tau pathology by downregulation of NMNAT1.

NMNAT enzyme activity in htau/mtau^+/−^ mice was similar to that of their control littermate mtau^+/^, while it was increased when NMNAT1 was overexpressed and decreased when NMNAT1 was downregulated, in agreement to corresponding changes in activity in *Nmnat1* tg and *Nmnat1*^+/−^ mice [22], [23]. Despite the modulation of NMNAT enzyme activity, which correlated to its levels of expression, steady-state NAD levels were not significantly different in all genotypes. Genetic overexpression of NMNAT in muscle was also found to have modest effects on NAD concentrations, which were thought to be maintained at a steady-state levels through a negative feedback mechanism [56]. We, however, cannot rule out that the increased NMNAT activity could cause localized changes in NAD availability and therefore affect the activity of downstream NAD dependent enzymes. In support of this, recent observations correlate nuclear NAD changes sensitive to the levels of NMNAT1 with sirtuin 1 (SIRT1), a NAD-dependent deacethylase, and disruption of nuclear-mitochondrial communication [47]. A number of mitochondrial proteins, involved in oxidative phosphorylation and other metabolic pathways were found altered in the cerebral cortices of 6 month old males 3xTg-AD which develop tau pathology as well as amyloid deposition [57]. Importantly, these alterations were found to precede amyloid deposition and neurofibrillary tangles formation, suggesting mitochondrial dysregulation as an early event in the pathogenesis of Alzheimer’s disease [58]. Although muscle data failed to observe alterations in mitochondrial NAD content following genetic overexpression of NMNAT [56], possible changes in mitochondrial homeostasis amongst the different NMNAT1 genotypes would support the hypothesis of localized variations of NAD availability and could be associated with more subtle neurodegenenerative changes. This will need to be investigated in further studies.

### 4.1 The protective effects of NMNAT1 overexpression in htau mice could be mediated by SIRT1, regardless of changes in NAD concentrations

SIRT1 is ubiquitously present in areas of the brain especially susceptible to age-related neurodegenerative states (e.g., the prefrontal cortex, hippocampus and basal ganglia) [59] and was found able to reduce the level of tau aggregates by promoting deacetylation and clearance of toxic tau species, a mechanism thought to be dependent upon nuclear NAD availability [60]. But SIRT1 activation could also be mediated by an increase in the cellular ratio of NAD to its reduced form NADH [56], [61] which is known to occur in response to an increased in NMAPT expression and activity [62] and can be associated with changes in enzymatic activity independently from changes in NAD concentrations [61], [62].

SIRT1 may also be involved in the delayed protection by NMNAT1 overexpression of the food burrowing deficit characteristic of htau mice. While NMNAT1 is located into the nucleus [7], SIRT1 shows dynamic changes in its subcellular localization during development and as a function of stress status [63]. In the mouse heart, SIRT1 is exclusively expressed in the nucleus at embryonic stages, but found in both the nucleus and cytoplasm at adulthood [64]. Although SIRT1 subcellular localization remains predominantly nuclear in the adult healthy brain [59], where it facilitates tau clearance [60], this redistribution could contribute to onset of tau pathology and associated early deficits in food burrowing behavior. Cellular stress, as is induced by tau accumulation [65], was found to trigger nuclear transport of SIRT1 [63], as well as alternative splicing of neuronal NMNAT towards the cytoplasmic variant which has robust neuroprotective capacity [66]. As such, the onset of tau pathology could switch NMNAT1 towards enhanced neuroprotective functions, in part *via* dynamic changes in SIRT1 subcellular localization. This mode of action could also possibly explain the differential effects of NMNAT1 downregulation on phosphorylated tau levels in the hippocampus and cerebral cortex, in which tau pathology develops at a different rate as. Insufficient nuclear SIRT1 levels is a possible mechanism whereby reducing NMNAT1 level by half could accelerate the onset of tau hyperphosphorylation. Our hippocampus data are indeed in agreement with previous observation in a Drosophila model of tauopathy in which it was found that downregulation of NMNAT accelerates the development of tau pathology [18]. It is however unclear why downregulation of NMNAT1 attenuated tau phosphorylation in the cortex, where the pathology developed earlier. Although one limitation of our study is that we only assessed tau hyperphosphorylation using AT8 immunoblotting, it is possible that reducing NMNAT1 level by half is not sufficient to prevent a neuroprotective response to tau accumulation when the pathology progresses. Altogether, this suggests that neuroprotective effects of NMNAT1 on behavioral symptoms, and possibly tau pathology, would only occur after disease onset, as was also seen in a Drosophila model of tauopathy [18]. The implication of SIRT1 in the age-dependency of the beneficial effects of nmnat1will need to be addressed in future studies.

In addition, protective effects of NMNAT could also be linked to its chaperone-like activity as seen in models a of polyQ toxicity [64] and in a Drosophila model of tauopathy where the chaperone function of NMNAT was found to contribute to tau clearance [18]. A similar explanation was proposed for the neuroprotective effect of lentiviral expression of NMNAT1 or NMNAT2 in the hippocampus of a mouse model of tauopathy [19].

## 5 Conclusion

In summary, we show that the reported early, specific behavioral deficit in food burrowing observed in htau mice [24] can be alleviated when NMNAT1 is overexpressed. The capability of NMNAT1 to notably ameliorate the food burrowing performance in the htau mouse model at very early stage highlights the potential for NMNAT1 to be a target for early stage AD and other pathologies, but the underlying mechanisms need to be addressed in future studies.

## Authors contribution

LC and MCP conceived and planned the study; LC, FR, PCG and MCP designed most experiments, with input from HF and MP; FR, PCG and WM performed most experiments; AHS, AL, MP, MRB and MYL conducted some experiments; data were collected and analyzed by FR, PCG and MRB, with input from WM, MYL and HF. LC and MCP supervised and coordinated the research with help from HF. FR, MCP and LC co-wrote the manuscript with additional input from PCG.
