# Supplementary material for: NAD-biosynthetic enzyme NMNAT1 reduces early behavioral impairment in the htau mouse model of tauopathy
Source: Behav Brain Res. 2018 Feb 26;339:140–52. doi: 10.1016/j.bbr.2017.11.030 (PMC5769520; doi:10.1016/j.bbr.2017.11.030)
Supplement: Supplementary file 1 [file mmc1.pdf]

SUPPLEMENTARY INFORMATION

NAD-biosynthetic enzyme NMNAT1 reduces early behavioral impairment in the htau mouse model of tauopathy

Francesca Rossi[1,2], Philippine C. Geiszler[1],Weina Meng[1], Matthew R. Barron[1], Malcolm Prior[2], Anna Herd-Smith[1], Andrea Loreto[1], Maria Yanez Lopez[3,4], Henryk Faas[3], Marie-Christine Pardon[1#*] and Laura Conforti[1#]

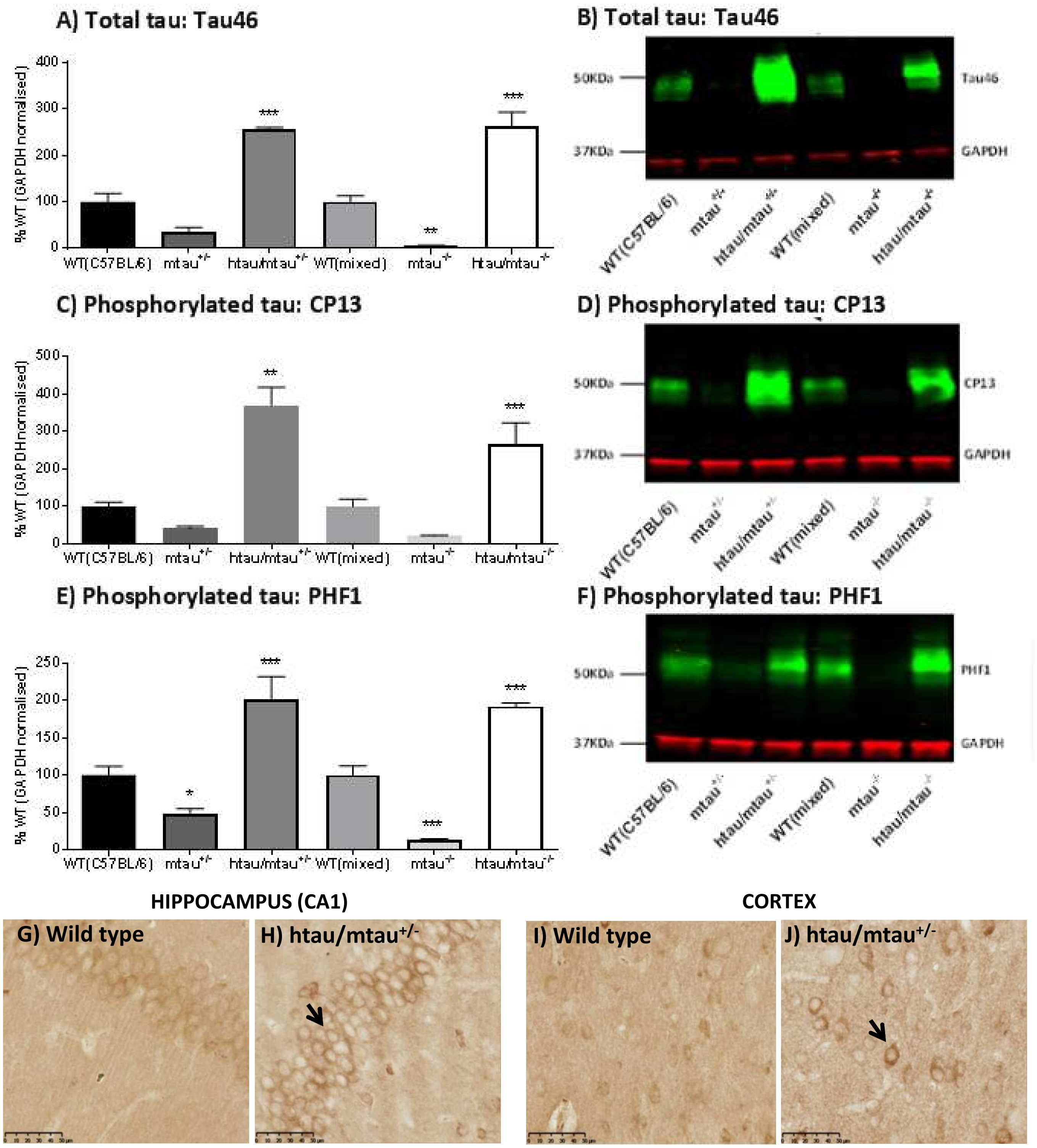

Suppl. Fig. 1: Tau pathology in 6-month-old htau mice heterozygous for murine tau. A-F) preliminary comparison of cortical tau levels in 6–month-old htau male mice on differing murine tau knockout background via western immunoblotting (n=3-4). mtau$^{+/-}$ and htau/mTau$^{+/-}$ mice were bred on a mixed genetic background and compared to their WT littermates. mtau$^{-/-}$ and htau/mtau$^{-/-}$ and were bred on a C57BL/6 background and compared to C57BL/6 controls. htau/mtau$^{+/-}$ and htau/mtau$^{-/-}$ mice displayed similarly elevated total (A and B) and phosphorylated (C-F) tau levels. *p<0.05, **p<0.01 and ***p<0.001 compared to corresponding WT. **G-J**) Hyperphosphorylated tau distribution in 6-month-old htau/mtau$^{+/-}$ mice. CP13 staining in the hippocampus and cortex shows higher accumulation of phosphorylated tau in neuronal cell bodies of htau/mtau$^{+/-}$ (H and J, black arrows) compared to wild type(G and I) mice. Scale bar: 50µm.

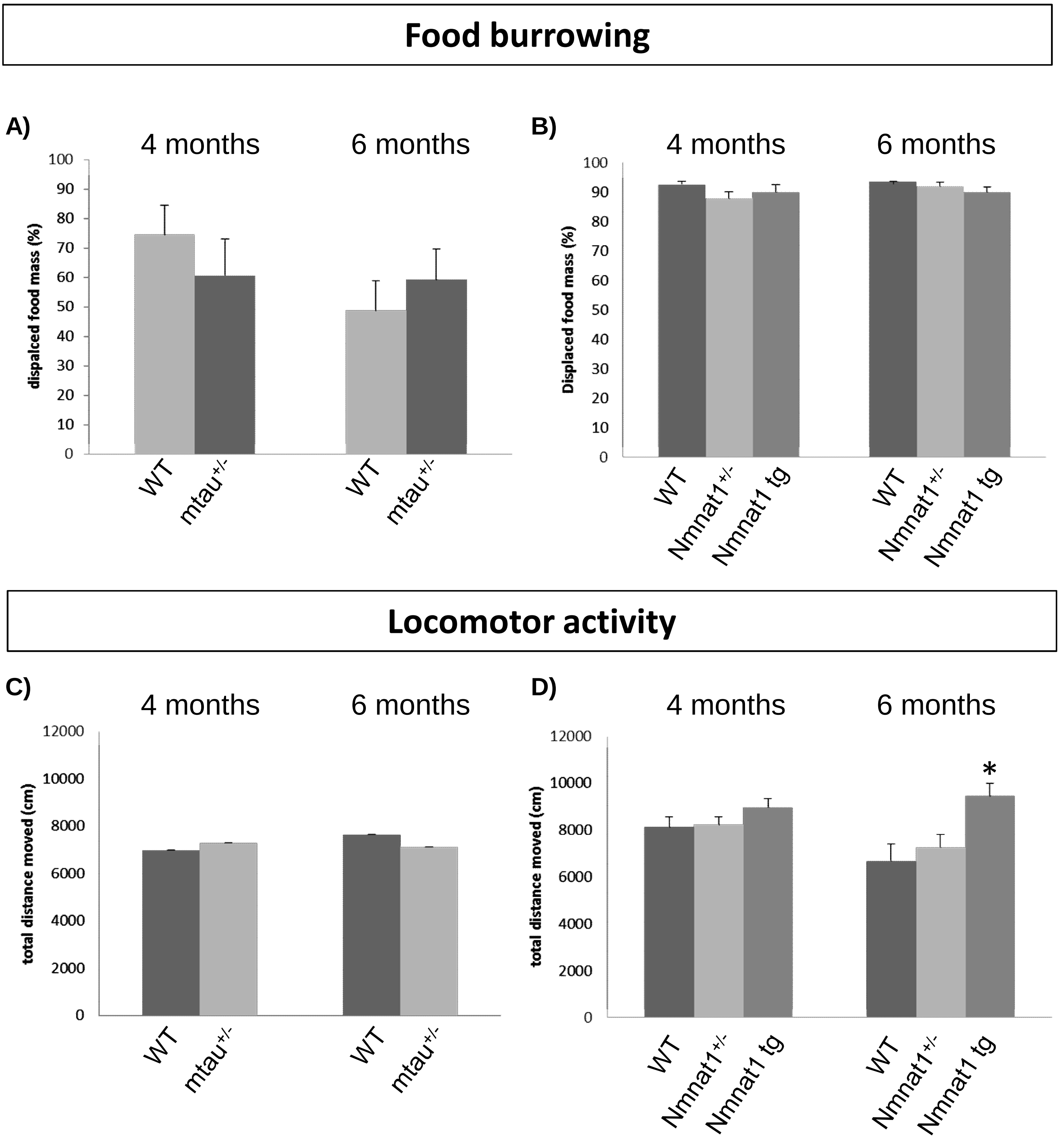

Suppl. Fig.2. Unaltered food burrowing and locomotor activity performance in *Nmnat1*$^{+/-}$, *Nmnat1* tg and *murine tau*$^{+/-}$ mice. Mice were tested at 4 and 6 months of age. Food burrowing performance was not altered neither in *murine tau*$^{+/-}$ mice (A) nor in *Nmnat1*$^{+/-}$ and *Nmnat1* tg mice (B) compared to their controls. Notes that in A) 50 grams of crushed food was used for food burrowing, whereas 30 grams was used in all other experiments. Locomotor activity was unaltered in *murine tau*$^{+/-}$ at 4 and 6 months of age compared to their wild type (C). *Nmnat1* tg mice showed a higher locomotor activity compared to the other genotypes at 6 months, but not at 4 months of age (D). WT = wild type; mtau$^{+/-}$ = *murine tau*$^{+/-}$. * $p<0.05$ compared to WT mice (n= 12/genotype).

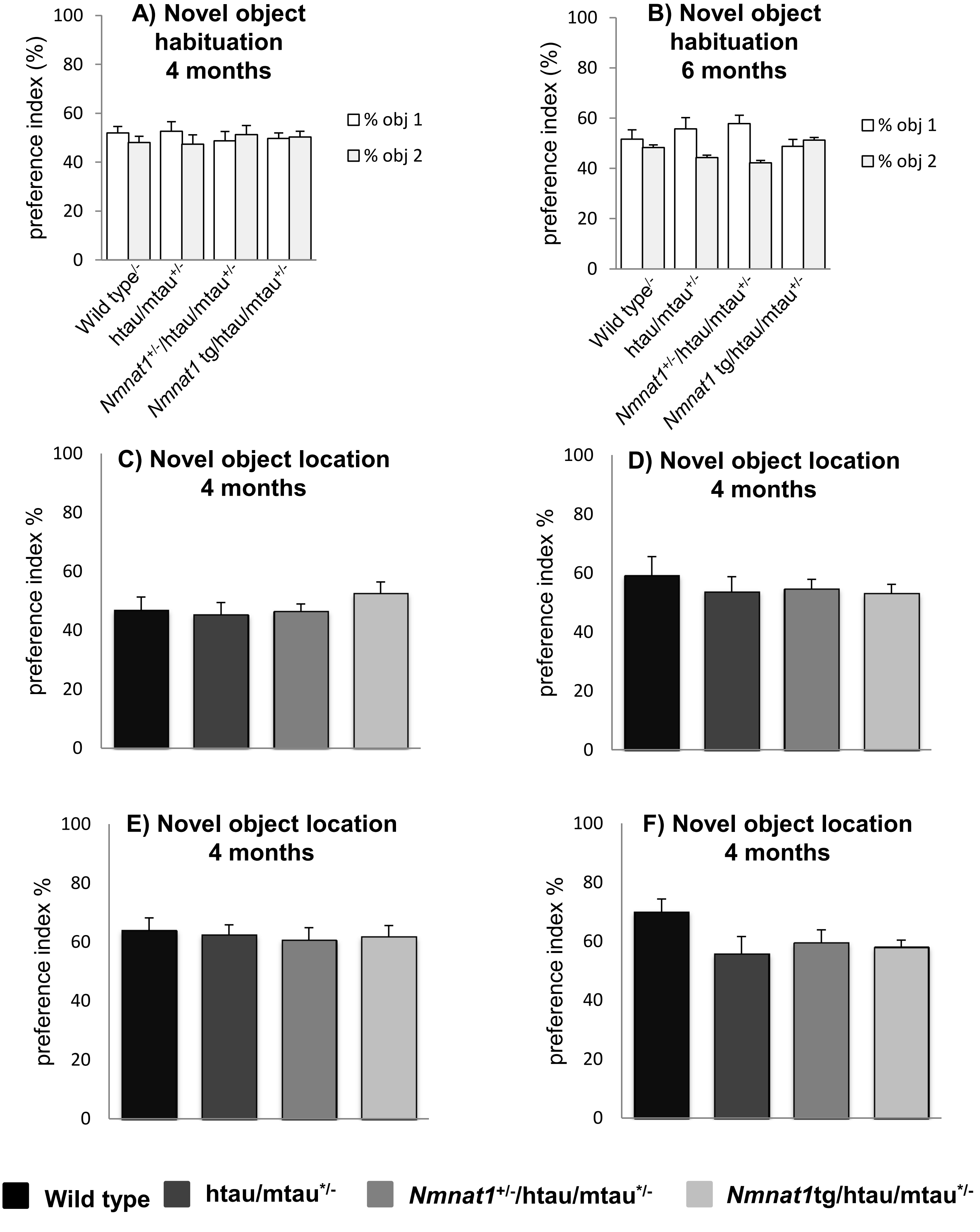

Suppl. Fig. 3. Novel object paradigm in htau mice. Mice were habituated to the objects and tested at 4 (A, B and C) and 6 months of age (D, E and F). No significant difference across genotypes was detected in memory location (B and E) and recognition performance (C and F). n = 12/group, Mean ± SEM.

Supplementary table 2. Results of the ANOVA tests on behavioral measures in mtau$^{+/-}$, *Nmnat1* tg and *Nmnat*$^{+/-}$ mice.

	Num. df	Den. df	F-value	p-value
Food burrowing test in Nmnat1tg and Nmnat1$^{+/-}$				
**sex**	1	25	2.23	0.148
**genotype**	2	25	1.54	0.234
**age**	1	25	1.27	0.27
**sex X genotype**	2	25	0.76	0.479
**sex X age**	1	25	0.12	0.734
**genotype X age**	2	25	0.6	0.558
**sex X genotype X age**	2	25	0.68	0.514
Open field (locomotor activity)in Nmnat1tg and Nmnat1$^{+/-}$				
**sex**	1	25	5.66	0.025
**genotype**	2	25	6.45	0.006
**age**	1	23	2.84	0.106
**sex X genotype**	2	25	0.86	0.437
**sex X age**	1	23	1.6	0.219
**genotype X age**	2	23	2.43	0.11
**sex X genotype X age**	2	23	0.65	0.534
Food burrowing test in mtau$^{+/-}$ mice				
**age**	2	5103.4	3.52	0.0354
**genotype**	1	12.22	0.01	0.9271
**age X genotype**	2	982.69	0.68	0.5113
Open field (locomotor activity) in mtau$^{+/-}$				
**age**	1	0.66	0.19	0.6621
**genotype**	1	0.38	0.11	0.7398
**age X genotype**	1	2.26	0.66	0.4193
